# Microsporidial Keratoconjunctivitis

**DOI:** 10.18502/jovr.v15i2.6746

**Published:** 2020-04-06

**Authors:** R Balamurugan, Parul Chawla Gupta, Surya Prakash Sharma, Neeti Rana, Jagat Ram

**Affiliations:** ^1^ Department of Ophthalmology, Post Graduate Institute of Medical Education and Research, Chandigarh, India

##  PRESENTATION

We report microsporidial keratoconjunctivitis in a young male exposed to muddy water while repairing a water distribution pipe successfully treated with topical voriconazole.

**Figure 1:**
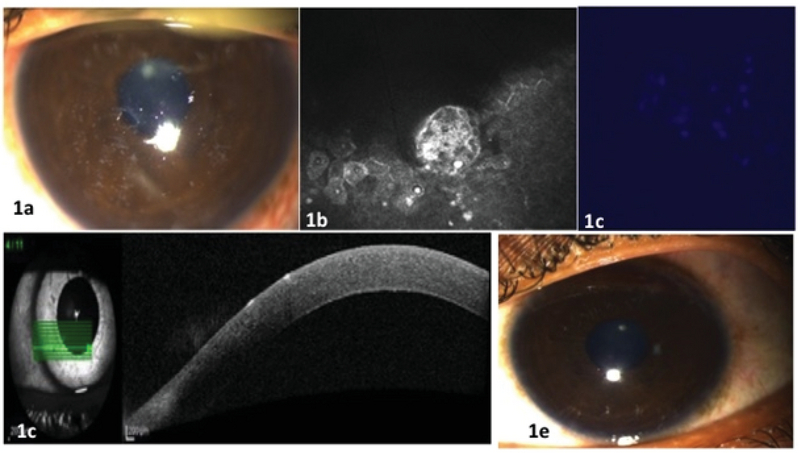
(a) Multifocal, coarse, white corneal epithelial infiltrates (b) In vivo confocal microscopy showing epithelial rosettes and intraepithelial pin–point hyperreflective spores (c) Combined potassium hydroxide-calcofluor white staining showing oval bluish fluorescent bodies (d) Anterior segment optical coherence tomography demonstrating stuck on plaques on the corneal surface (e) Slit lamp biomicroscopy showing clear cornea after treatment.

##  DISCUSSION

A 22-year-old healthy male who is a plumber by profession presented with chief complaints of sudden pain, redness, watering, and decreased vision in his left eye. These symptoms occurred two days after a water distribution pipe that he was repairing burst and the water came in contact with his eye. A slit-lamp examination of his left eye revealed multifocal, coarse, white corneal epithelial infiltrates [Figure 1(a)]; he had a visual acuity of 20/200. *In vivo* confocal microscopy revealed epithelial rosettes and intraepithelial pinpoint hyper-reflective spores [Figure 1(b)]. Staining with potassium hydroxide–calcofluor white revealed oval bluish fluorescent bodies [Figure 1(c)]. Anterior segment optical coherence tomography demonstrated stuck-on-plaques on the surface of the cornea [Figure 1(d)]. Slit-lamp biomicroscopy revealed a clear cornea after hourly treatment with voriconazole drops. After one week of treatment, the cornea was clear and visual acuity improved to 20/20 [Figure 1(e)].

Microsporidial keratoconjunctivitis occurs primarily in males and is usually unilateral. It has recently been observed to cause multifocal coarse punctate epithelial keratitis in immunocompromised individuals, In addition to the use of contact lenses, another risk factor for microsporidial keratoconjunctivitis that may be less recognized because of the lack of awareness among ophthalmologists is the exposure to muddy water^[2,3]^ predominantly observed during the rainy season^[4]^ in developing countries.

##  Financial Support and Sponsorship

None.

##  Conflicts of Interest

There are no conflicts of interest.
